# Supplementary material for: Deep learning classification of shoulder fractures on plain radiographs of the humerus, scapula and clavicle
Source: PLoS One. 2023 Aug 30;18(8):e0289808. doi: 10.1371/journal.pone.0289808 (PMC10468075; doi:10.1371/journal.pone.0289808)
Supplement: S1 Checklist — (DOCX) [file pone.0289808.s001.docx]

**CLAIM: Checklist for Artificial Intelligence in Medical Imaging**

| Section / Topic | No. | Item |  |
| --- | --- | --- | --- |
| TITLE / ABSTRACT |  |  |  |
|  | **1** | Identification as a study of AI methodology, specifying the category of technology used (e.g., deep learning) | **1** |
|  | **2** | Structured summary of study design, methods, results, and conclusions | **2** |
| INTRODUCTION |  |  |  |
|  | **3** | Scientific and clinical background, including the intended use and clinical role of the AI approach | **3-4** |
|  | **4** | Study objectives and hypotheses | **4** |
| METHODS |  |  |  |
| *Study Design* | **5** | Prospective or retrospective study | **4** |
|  | **6** | Study goal, such as model creation, exploratory study, feasibility study, non-inferiority trial | **-** |
| *Data* | **7** | Data sources | **4** |
|  | **8** | Eligibility criteria: how, where, and when potentially eligible participants or studies were identified (e.g., symptoms, results from previous tests, inclusion in registry, patient-care setting, location, dates) | **4-5** |
|  | **9** | Data pre-processing steps | **-** |
|  | **10** | Selection of data subsets, if applicable | **-** |
|  | **11** | Definitions of data elements, with references to Common Data Elements | **-** |
|  | **12** | De-identification methods | **4** |
|  | **13** | How missing data were handled | **-** |
| *Ground Truth* | **14** | Definition of ground truth reference standard, in sufficient detail to allow replication | **5-8** |
|  | **15** | Rationale for choosing the reference standard (if alternatives exist) | **-** |
|  | **16** | Source of ground-truth annotations; qualifications and preparation of annotators | **5** |
|  | **17** | Annotation tools | **5** |
|  | **18** | Measurement of inter- and intrarater variability; methods to mitigate variability and/or resolve discrepancies | **13** |
| *Data Partitions* | **19** | Intended sample size and how it was determined | **-** |
|  | **20** | How data were assigned to partitions; specify proportions | **4** |
|  | **21** | Level at which partitions are disjoint (e.g., image, study, patient, institution) | **-** |
| *Model* | **22** | Detailed description of model, including inputs, outputs, all intermediate layers and connections | **8** |
|  | **23** | Software libraries, frameworks, and packages | **8-9** |
|  | **24** | Initialization of model parameters (e.g., randomization, transfer learning) | **-** |
| *Training* | **25** | Details of training approach, including data augmentation, hyperparameters, number of models trained | **8** |
|  | **26** | Method of selecting the final model | **-** |
|  | **27** | Ensembling techniques, if applicable | **-** |
| *Evaluation* | **28** | Metrics of model performance | **8-9** |
|  | **29** | Statistical measures of significance and uncertainty (e.g., confidence intervals) | **-** |
|  | **30** | Robustness or sensitivity analysis | **-** |
|  | **31** | Methods for explainability or interpretability (e.g., saliency maps), and how they were validated | **-** |
|  | **32** | Validation or testing on external data | **-** |
| RESULTS |  |  |  |
| *Data* | **33** | Flow of participants or cases, using a diagram to indicate inclusion and exclusion | **-** |
|  | **34** | Demographic and clinical characteristics of cases in each partition | **-** |
| *Model performance* | **35** | Performance metrics for optimal model(s) on all data partitions | **9-13** |
|  | **36** | Estimates of diagnostic accuracy and their precision (such as 95% confidence intervals) | **9-13** |
|  | **37** | Failure analysis of incorrectly classified cases | **-** |
| DISCUSSION |  |  |  |
|  | **38** | Study limitations, including potential bias, statistical uncertainty, and generalizability | **14-16** |
|  | **39** | Implications for practice, including the intended use and/or clinical role | **16** |
| OTHER INFORMATION |  |  |  |
|  | **40** | Registration number and name of registry | **-** |
|  | **41** | Where the full study protocol can be accessed | **-** |
|  | **42** | Sources of funding and other support; role of funders | **-** |

Mongan J, Moy L, Kahn CE Jr. Checklist for Artificial Intelligence in Medical Imaging (CLAIM): a guide for authors and reviewers. Radiol Artif Intell 2020; 2(2):e200029. <https://doi.org/10.1148/ryai.2020200029>
