# Supplementary material for: Deep learning classification of shoulder fractures on plain radiographs of the humerus, scapula and clavicle
Source: PLoS One. 2023 Aug 30;18(8):e0289808. doi: 10.1371/journal.pone.0289808 (PMC10468075; doi:10.1371/journal.pone.0289808)
Supplement: Attachment — Submitted filename: Response to reviewers.docx [file pone.0289808.s002.docx]

Dear Professor Simmen,

I appreciate the opportunity to revise our manuscript entitled "Deep learning classification of shoulder fractures and dislocations on plain radiographs of the humerus, scapula and clavicle" for potential publication in PLOS ONE. My team and I are grateful for the reviewers' constructive feedback, and we believe that their comments have significantly improved our paper. We have revised the manuscript addressing each point raised by the reviewers and have provided a detailed response to the comments below.

**Reviewer 1 Comments**

1) General comment
The authors show in their study “Deep learning classification of shoulder and dislocations on plain radiographs of the humerus, scapula and clavicle” that their deep learning model based on a convolutional neural network (CNN) perfom promising results in fracture-classification and degenerative conditions.
I guess the artificial intelligence (AI) is coming a key player in prevention, diagnosis, treatment, patient monitoring and data management in medicine. Therefore machine learning/neuronal networks and deep neuronal networks are very important methodical approaches in the future. The applied CNN is one type of deep neuronal networks which based on different hidden layers with billions of nodes and is a further development of neuronal networks. In this way CNN are used in recognizing of images.
All in all, it is an interesting study about fracture classification based on artificial intelligence using a CNN. An overall area under the curve (AUC) has a good performance for fracture classification related to proximal and diaphyseal humerus fractures. Additionally the authors show good to excellent AUC for clavicule, and scapula fractures.
Overall this study is a good example as the algorhythms of deep neuronal networks can work with unstructured data without processing to structured data. The algorhythms are extracting itself the essential characteristics to differentiate the data. Finally, this CNN model recognize patterns and interpret the variations.
The demonstrated application of AI in fracture classification is a very interesting example in medicine. And the possible applications will probably expand to other medical fields.

**Response:** Thank you for your thoughtful and thorough review of our study. We appreciate your recognition of the potential of AI and our work in the field of fracture classification using CNNs. We are encouraged by your positive feedback and will continue to explore the potential applications of AI in medical fields.

1) "The study conducted by the authors focuses on applying CNN to fracture-classification and not to degenerative bone changes. So I would omit the remarks about degenerative conditions of bone."

**Response:** We appreciate your suggestion and agree that this might confuse the reader. To better represent our study focus, removed references to other labelled findings such as degenerative conditions. The removed sections were found on lines: 33, 97-98 in the first submission. We have removed reference number 13 on shoulder dislocations and updated the reference list, so it excludes this reference.

2) "The topic of shoulder dislocation injuries does not fit into the topic of fracture classifications generated using deep neuronal networks (DNN)."

**Response:** We understand your concern. We aimed to demonstrate the versatility of our model, including its application to dislocation injuries. However, to maintain focus on fracture classification, we have removed all sections on anterior shoulder dislocations, and removed dislocations from the title. The removed sections were found on lines: 59-60, 70, 128-130, 225-228, and removed reference number 13 from the first submitted manuscript.

3) "The classification of dislocation injuries of acromioclavicular joints is missing from Table 1. But I would omit it altogether anyway as this entity does not contribute anything significant to the main topic."

**Response:** We apologize for the oversight. However, following your valuable suggestion, we have now omitted dislocation injuries from our study to maintain focus on fracture classifications.

4) "This sentence is misleading. A diagnostic tool should generate a diagnosis that can be used a decision-making aid in order to make a possible indication for surgery."

**Response:** We agree with your comment. We did not mean to suggest that AI would replace clinical decision-making. Rather, we wanted to highlight that AI could assist in classifying fractures more objectively, potentially leading to more uniform treatment strategies. We have revised the sentence to avoid any confusion. The revised text is found on line xxx

**Reviewer 2 Comments**

1) "The upbringing of shoulder dislocations comes quite surprisingly at this point."

**Response:** We agree with your feedback and have now revised the introduction to provide a clearer picture of our study focus on fracture classifications, and removed all sections on dislocations (please see response to reviewer 1).

2) "This sentence is not supported by the data provided."

**Response:** We appreciate your critique and agrees with you that statement was not supported by the data. We have now revised the discussion to more accurately reflect the findings of our study. The revised text is found on lines 277-280.

**Journal Requirements**

1) We have ensured that our manuscript now follows PLOS ONE's style requirements.

2) We have updated the Data Availability statement to specify where the minimal dataset can be found. We will also include URLs or DOIs for the data in the cover letter.

3) We have obtained written permission from the copyright holder to publish Figures 1 and 2 under the CC BY 4.0 license. This documentation will be uploaded as an "Other" file with our submission.

4) We have reviewed our reference list and confirmed that it is complete and correct.

We sincerely thank you for considering our manuscript for publication in PLOS ONE. We believe the revised manuscript has addressed the concerns raised and hope that it is now acceptable for publication.

Best Regards,

Martin Magnéli, MD, PhD
